# The associations between interleukin-17 single-nucleotide polymorphism and colorectal cancer susceptibility: a systematic review and meta-analysis

**DOI:** 10.1186/s12957-022-02586-2

**Published:** 2022-04-12

**Authors:** Gaoming Li, Jingfu Ma, Ning Zhang, Xiaogang Li, Fangfang Li, Yuxing Jiang

**Affiliations:** 1 Center for Disease Control and Prevention of Central Theater Command, Shijingshan District, Beijing, China; 2 Department of Intensive Care Unit, The 305 Hospital of PLA, Beijing, China; 3 Department of General surgery, The 305 Hospital of PLA, Beijing, China

**Keywords:** Colorectal cancer, IL-17, Polymorphism, Susceptibility, Meta-analysis

## Abstract

**Background:**

Numerous case-control studies have reported associations between interleukin-17 (IL-17) polymorphisms and colorectal cancer; however, the results were inconsistent. The aim of this meta-analysis was to further clarify the effects of IL-17 polymorphisms on colorectal cancer susceptibility.

**Materials and method:**

Relevant studies were extracted from the electronic databases PubMed, Embase, Web of Science, China National Knowledge Infrastructure (CNKI), and the Chinese Biomedical Literature Database (CMB) up to April 2021. The odds ratio and 95% confidence interval were used to estimate the strength of the associations.

**Results:**

Ten articles including 2599 cases and 2845 controls were enrolled in our research after strict literature screening. Highly significant associations between the IL-17A rs2275913 polymorphism and increased colorectal cancer susceptibility were observed in all five gene models (allelic, dominant, recessive, homozygous, and heterozygous models), and subgroup analysis based on ethnicity revealed that these associations existed not only in the Asian population but also in the Caucasian population. However, the results showed no significantly elevated colorectal cancer risk correlated with the IL-17F rs763780 polymorphism, and a slightly lower colorectal cancer susceptibility for the Caucasian population was discovered in the recessive and homozygous models of this mutation.

**Conclusion:**

The IL-17A rs2275913 polymorphism may be an independent risk factor contributing to colorectal cancer susceptibility, while the IL-17F rs763780 polymorphism may decrease susceptibility to colorectal cancer. Future studies with large-scale samples are warranted to identify these associations.

**Supplementary Information:**

The online version contains supplementary material available at 10.1186/s12957-022-02586-2.

## Introduction

Epidemiological data from the last year showed that colorectal cancer has become the third most common and the second most lethal malignant tumor. With a high morbidity and mortality, colorectal cancer causes almost 2 million diagnosed cases and approximately 1 million cancer-related deaths throughout the world per year [1], posing a major threat to normal life and imposing a heavy global burden on human health [2]. Although the specific mechanism of colorectal cancer tumorigenesis remains uncertain, accumulative evidence has demonstrated that factors, such as the environment, diet, smoking, alcohol, and some precancerous lesions, are closely associated with the occurrence of colorectal cancer [3–5]. However, even if exposed to the same environmental factors, only a small proportion of people suffer from colorectal cancer, which suggests that genetic factors might play a crucial role in the pathogenesis of colorectal cancer. Some current studies have indicated that single-nucleotide polymorphisms, especially polymorphisms from inflammatory cytokines, interfere with and modify protein expression and increase colorectal malignant tumor susceptibility [6, 7].

The synergy of the tumor microenvironment and some inflammatory cytokines is well recognized in cancer progression [8, 9]. Chronic inflammation has been proven to be strongly associated with genetic instability and related mechanisms in the cancer inflammatory microenvironment, indicating that gene mutation and inflammation may closely participate in the pathogenesis and progression of malignant tumors [10]. IL-17, also called IL-17A or CTLA-8, is an inflammatory cytokine secreted by T-helper 17 cells. As the named subspecies in the IL-17 gene family, it was first discovered from the cDNA of hybrid rodent T cells [11]. The IL-17 family contains at least six members, IL-17A to F, with all of them having similar gene sequences and biological functions [12]. Recently, a number of studies have confirmed the effects of IL-17 on the initiation and development of multiple types of malignancies, including hepatocellular carcinoma [13], lung cancer [14], pancreatic cancer [15], and cervical cancer [16]. Although IL-17A and IL-17F were clarified as risk factors for colorectal cancer during the most recent decades [17], the concrete reasons were unclear.

It is widely reported that IL-17A and IL-17F polymorphic variants are correlated with increased susceptibility to several digestive system primary malignancies, such as gastric cancer [18], esophageal cancer [19], hepatocellular carcinoma [20, 21], and oral squamous cell carcinoma [22]. Additionally, some scholars proposed that the IL-17F polymorphism might confer poor survival for advanced pancreatic cancer patients [23]. Positive relationships were observed between inflammatory bowel diseases, regarded as precancerous lesions of colorectal cancer, and IL-17A and IL-17F polymorphisms in several previous studies [24, 25], and an increasing number of studies have been performed to investigate whether these polymorphisms contribute to colorectal cancer; however, the results were still inconclusive. Hence, this meta-analysis was conducted to first explore the association between IL-17A rs2275913 and IL-17F rs763680 polymorphisms and colorectal cancer.

## Materials and methods

### Search strategy for the literature

An Internet search for the literature published in English or Chinese was conducted from the establishment date of the PubMed, Web of Science, Embase, CNKI, and CMB databases to April 2021, with the following keywords: “interleukin-17 or IL-17 or CTLA-8,” “CRC or colorectal cancer or colon cancer or rectal cancer,” and “SNP or polymorphism or single-nucleotide polymorphism or gene mutation or gene variant.” Relevant conference papers were retrieved using the journal database of the National Library of China by a manual search.

### Inclusion and exclusion criteria

All of the eligible studies included in this meta-analysis met the following criteria:

1. The studies were set out to investigate the associations between IL-17A rs2275913 or IL-17F rs763780 polymorphisms and colorectal cancer susceptibility.
2. The studies were case-control studies.
3. There were available and adequate genotype frequencies to evaluate the odds ratio (OR) and 95% confidence interval (CI).
4. The studies were carried out only on human beings.

The studies with the following criteria were excluded from this meta-analysis:

1. The aims of the studies were not to detect the effect of IL-17A rs2275913 or IL-17F rs763780 polymorphism on colorectal cancer.
2. Non-case-control studies
3. Duplicated publications or studies with overlapping data
4. The studies without extractable data of genotype frequencies
5. The publications were identified as reviews, case reports, letters to editors, and brief communications.

### Data extraction

Available data were extracted by two independent investigators from the enrolled articles, including the study author, study year, study design, ethnicity of population, source of controls, genotyping methods, matching criteria of cases and controls, genotype frequencies, and the calculated Hardy–Weinberg equilibrium (HWE). For repeated publications, only the studies with the largest sample size and highest quality or the most exhaustive information were selected. If any disagreement appeared, a third investigator was involved in the discussion until a final agreement was reached.

## Quality score assessment

The quality of each enrolled study was assessed by the developed standard consisting of 6 aspects of representativeness of cases, source of controls, case-control matching, specimens used for determining genotypes, HWE, and total sample size as previously reported (Table 1) [26]. The total score ranged from 0 to 18, and the score for each aspect ranged from 0 to 3. Literature with a total score ≥ 12 was considered high quality; otherwise, literature with a total score < 12 was considered low quality.

**Table 1:** The criteria list of quality score for included studies

Criterion	Score
**Representativeness of cases**	
Selected from population or cancer registry	3
Selected from hospital	2
Selected from pathology archives, but without description	1
Not described	0
**Source of controls**	
Population based	3
Blood donors or volunteers	2
Hospital based (cancer-free patients)	1
Not described	0
**Case-control match**	
Matched by age and gender	3
Not matched by age and gender	0
**Specimens used for determining genotypes**	
White blood cells or normal tissues	3
Tumor tissues or exfoliated cells of tissue	0
**Hardy–Weinberg equilibrium (HWE)**	
Hardy–Weinberg equilibrium in control subjects	3
Hardy–Weinberg disequilibrium in control subjects	0
**Total sample size**	
> 1000	3
> 500 and < 1000	2
> 200 and < 500	1
< 200	0

## Statistical analysis

All statistical tests in this study were bilateral, and differences with *P* < 0.05 were considered statistically significant unless otherwise stated. The association of mutation sites with colorectal cancer risk was assessed by the odds ratio (OR) and its corresponding 95% confidence interval (CI), and the *Z*-test was used for the statistical significance test of the combined OR value. The *χ*^2^ test was used to test whether the genotypes of the control group met HWE. The Cochrane *Q*-test was used to detect whether heterogeneity existed among the studies, and its statistical quantity *Q* approximately followed the *χ*^2^ distribution with k-1 degrees of freedom (k was the number of studies). A *P*-value less than 0.10 suggested that heterogeneity existed among studies. At the same time, heterogeneity was quantitatively evaluated by combining the *I*^2^ values. The *I*^2^ value ranged from 0 to 100%, and the larger the value was, the higher the heterogeneity. In general, *I*^2^ less than 25% indicated mild heterogeneity, *I*^2^ between 25 and 50% indicated moderate heterogeneity, and *I*^2^ more than 50% indicated high heterogeneity. When the heterogeneity test in various studies was *P* < 0.10 or *I*^2^ > 50%, the random effect model (DerSimonian–Laird method) was employed for meta-analysis; otherwise, the fixed effect model (Mantel–Haenszel method) was employed. Sensitivity analysis was performed to determine the stability of conclusions by removing the enrolled studies one by one and estimating whether the results changed. The funnel plots drawn by effect size and standard error were carried out to evaluate possible publication bias, and Begg’s rank correlation was used to test the asymmetry of the funnel plots. All statistical analyses were calculated using Stata version 13.0 software (STATA Corporation, College Station, TX, USA).

## Results

### Characteristics of publications

**Fig. 1:**
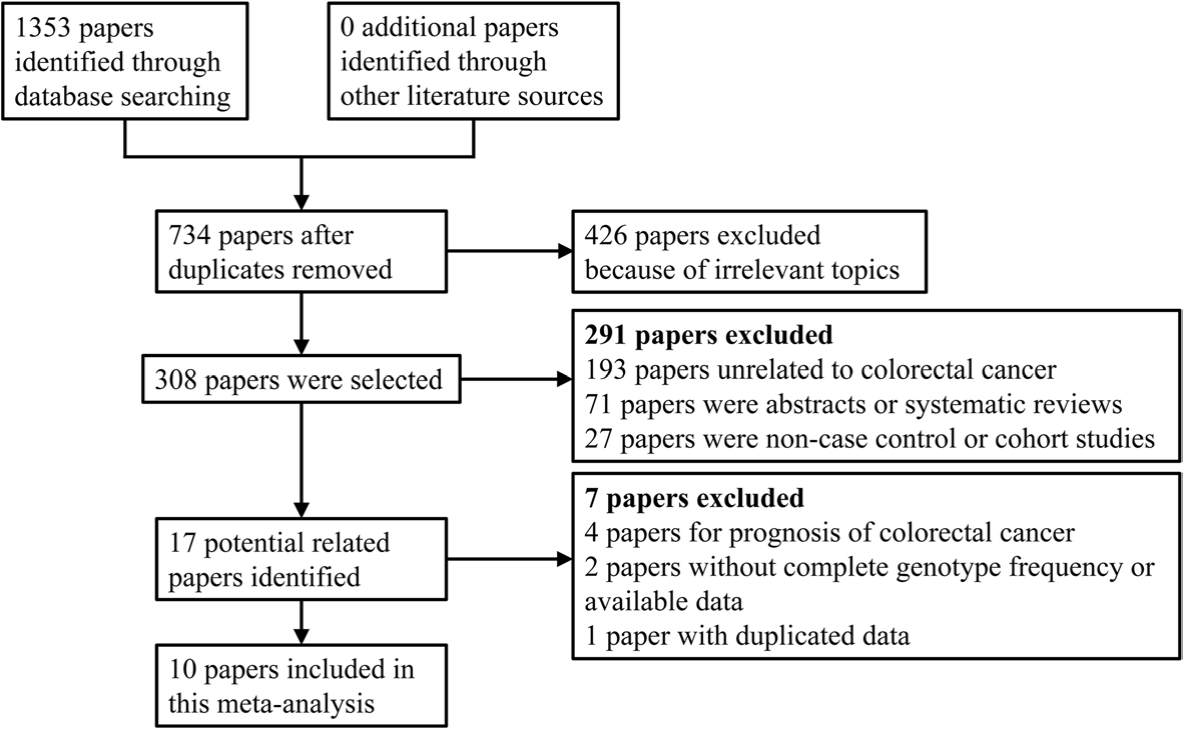
Flow diagram for the literatures included in this present meta-analysis

In total, 1353 related articles were obtained in the preliminary examination, and the remaining 619 articles were excluded from repeated articles. According to the inclusion and exclusion criteria, the preliminary screening for articles was conducted by reading titles and abstracts, and 426 articles unrelated to the research topic were excluded. After further reading the full text, 298 articles were excluded, including 193 studies unrelated to colorectal cancer, 71 abstracts or systematic reviews, 27 non-case-control or cohort studies, 4 prognostic studies of colorectal cancer, 2 without complete genotype frequency or available data, and 1 with duplicated data. Finally, 14 case-control studies, including 2599 cases and 2845 controls from 10 papers meeting the inclusion criteria, were selected for this meta-analysis [27–36] (Fig. 1). The first authors for each included paper were from 6 different nations, and the geographical areas consisted of East Asia, West Asia, and North Africa. The number of coauthors in each paper ranged from 2 to 13, and the researchers of 6 papers received explicit funding support. Among the included studies, 8 were conducted for the IL-17A rs2275913 polymorphism, and 6 were conducted for the IL-17F rs763780 polymorphism; meanwhile, 6 were conducted on Asians, and 8 were conducted on Caucasians. A total of 9 studies were considered high quality (≥ 12) via quality score assessment. The basic characteristics of each included study are summarized in Table 2.

**Table 2:** Basic characteristics of included studies for this meta-analysis

Study author	Study year	Country	Ethnicity	Cancer type	Design	Source of controls	Genotyping method	Matching criteria	Cases			Controls			HWE	Quality score
									AA	AG	GG	AA	AG	GG		
**IL-17A rs2275913**																
Omrane et al.	2014	Tunisia	Caucasian	Colorectal cancer	Retrospective study	HB	PCR-RFLP	Not mentioned	3 (2.9%)	51 (50.0%)	48 (47.1%)	6 (4.3%)	38 (27.4%)	95 (68.3%)	0.387	10
Nemati et al.	2015	Iran	Caucasian	Colorectal cancer	Retrospective study	PB	PCR-RFLP	Age, sex, ethnic, geographic origin	82 (27.0%)	100 (32.9%)	122 (40.1%)	50 (17.4%)	110 (38.2%)	128 (44.4%)	0.002	14
Samiei et al.	2018	Malaysia	Asian	Colorectal cancer	Retrospective study	PB	PCR-RFLP	Not mentioned	27 (38.6%)	33 (47.1%)	10 (14.3%)	12 (15.0%)	41 (51.2%)	27 (33.8%)	0.577	11
Al Obeed et al.	2018	Saudi Arabia	Caucasian	Colorectal cancer	Retrospective study	PB	qRT-PCR	Gender, age	17 (14.5%)	40 (34.2%)	60 (51.3%)	7 (7.0%)	23 (23.0%)	70 (70.0%)	0.018	12
Bedoui et al.	2018	Tunisia	Caucasian	Colorectal cancer	Retrospective study	PB	TaqMan	Ethnic origin	14 (4.8%)	79 (27.1%)	199 (68.1%)	9 (3.5%)	58 (22.2%)	194 (74.3%)	0.084	14
Feng et al.	2019	China	Asian	Colorectal cancer	Retrospective study	PB	PCR-RFLP	Sex, age	37 (10.5%)	154 (43.9%)	16 0 (45.6%)	31 (7.2%)	169 (39.2%)	231 (53.6%)	0.991	16
Moundir et al.	2019	Morocco	Caucasian	Colorectal cancer	Retrospective study	HB	TaqMan	Not mentioned	41 (58.6%)	22 (31.4%)	7 (10.0%)	27 (38.6%)	18 (25.7%)	25 (35.7%)	< 0.001	6
Zhang et al.	2020	China	Asian	Colorectal cancer	Retrospective study	PB	PCR-RFLP	Gender, age	41 (19.7%)	110 (52.9%)	57 (27.4%)	45 (14.4%)	149 (47.8%)	118 (37.8%)	0.854	16
**IL-17F rs763780**																
Omrane et al.	2014	Tunisia	Caucasian	Colorectal cancer	Retrospective study	HB	PCR-RFLP	Not mentioned	1 (0.7%)	38 (27.8%)	98 (71.5%)	1 (1.0%)	27 (27.0%)	72 (72.0%)	0.374	10
Nemati et al.	2015	Iran	Caucasian	Colorectal cancer	Retrospective study	PB	PCR-RFLP	Age, sex, ethnic, geographic origin	337 (93.6%)	0 (0.0%)	23 (6.4%)	391 (97.3%)	0 (0.0%)	11 (2.7%)	< 0.001	14
Li et al.	2016	China	Asian	Colorectal cancer	Retrospective study	PB	PCR-HRM	Sex, age	0 (0.0%)	13 (26.0%)	37 (74.0%)	0 (0.0%)	10 (20.0%)	40 (80.0%)	0.432	14
Samiei et al.	2018	Malaysia	Asian	Colorectal cancer	Retrospective study	PB	PCR-RFLP	Not mentioned	5 (7.2%)	25 (35.7%)	40 (57.1%)	1 (1.2%)	23 (28.8%)	56 (70.0%)	0.419	11
Al Obeed et al.	2018	Saudi Arabia	Caucasian	Colorectal cancer	Retrospective study	PB	qRT-PCR	Gender, age	110 (94.0%)	7 (6.0%)	0 (0.0%)	94 (94.0%)	6 (6.0%)	0 (0.0%)	0.757	15
Feng et al.	2019	China	Asian	Colorectal cancer	Retrospective study	PB	PCR-RFLP	Sex, age	10 (2.8%)	100 (28.5%)	241 (68.7%)	16 (3.7%)	132 (30.6%)	284 (65.7%)	0.892	16

### Associations between the IL-17A rs2275913 polymorphism and colorectal cancer

**Fig. 2:**
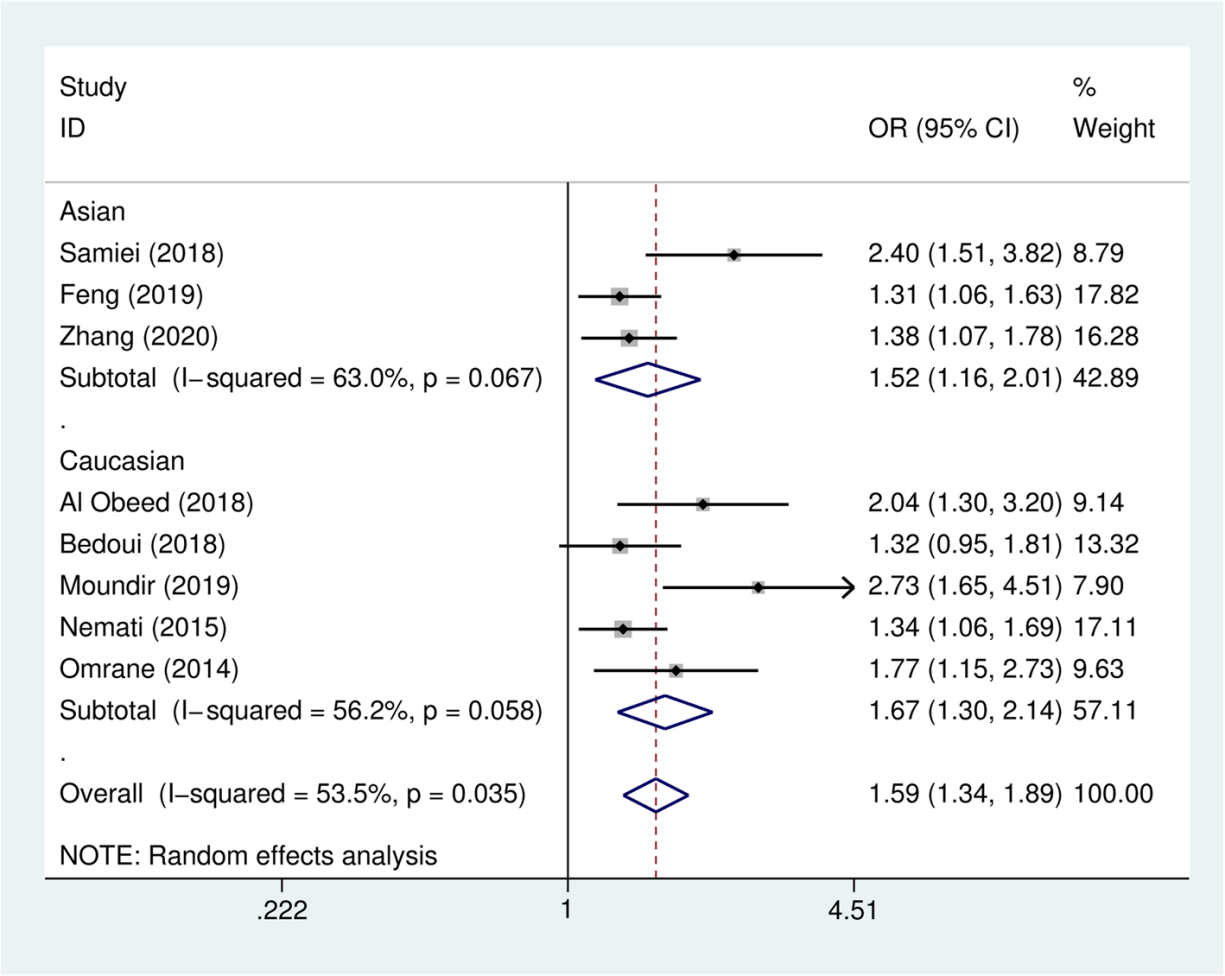
The forest plot of the allelic model (A vs. G) for the associations between IL-17A rs2275913 polymorphism and colorectal cancer. The study-specific ORs are represented as squares. The size of the square indicates the weight of each study. The horizontal lines represent 95% CIs. Diamonds show the overall estimate or pooled ORs in subgroups with their corresponding 95% CIs

Overall, the analysis revealed that all five genetic models (allelic, dominant, recessive, homozygous, and heterozygous models) of the IL-17A rs2275913 polymorphism were related to an elevated colorectal cancer risk (A vs. G: *OR* = 1.59, 95% *CI* = 1.34–1.89, *P* < 0.001; AA/AG vs. GG: *OR* = 1.75, 95% *CI* = 1.36–2.25, *P* < 0.001; AA vs. GG/AG: *OR* = 1.74, 95% *CI* = 1.41–2.15, *P* < 0.001; AA vs. GG: *OR* = 2.05, 95% *CI* = 1.62–2.60, *P* < 0.001; AG vs. GG: *OR* = 1.60, 95% *CI* = 1.23–2.09, *P* = 0.001) (Table 3). When subgroup analysis was performed according to ethnicity, a higher risk of colorectal cancer was observed not only in the Asian population (A vs. G: *OR* = 1.52, 95% *CI* = 1.16–2.01, *P* =0.003; AA/AG vs. GG: *OR* = 1.62, 95% *CI* = 1.18–2.23 *P* =0.003; AA vs. GG/AG: *OR* = 1.72, 95% *CI* = 1.26–2.34, *P* = 0.001; AA vs. GG: *OR* = 2.10, 95% *CI* = 1.49–2.96, *P* < 0.001; AG vs. GG: *OR* = 1.43, 95% *CI* = 1.14–1.80, *P* = 0.002) but also in the Caucasian population (A vs. G: *OR* = 1.67, 95% *CI* = 1.30–2.14, *P* < 0.001; AA/AG vs. GG: *OR* = 1.88, 95% *CI* = 1.26–2.81 *P* =0.002; AA vs. GG/AG: *OR* = 1.76, 95% *CI* = 1.32–2.36, *P* <0.001; AA vs. GG: *OR* = 2.01, 95% *CI* = 1.46–2.77, *P* < 0.001; AG vs. GG: *OR* = 1.76, 95% *CI* = 1.11–2.80, *P* = 0.017) (Fig. 2, Supplementary Fig. 1 A–D). The result from stratified analysis classified by the source of controls exhibited a significant colorectal cancer susceptibility correlated to IL-17A rs2275913 polymorphism in population-based (PB) (A vs. G: OR = 1.47, 95% CI = 1.25–1.72, *P* <0.001; AA/AG vs. GG: OR = 1.50, 95% CI = 1.23–1.83, *P* <0.001; AA vs. GG/AG: OR = 1.74, 95% CI = 1.39–2.18, *P* <0.001; AA vs. GG: OR = 1.96, 95% CI = 1.53–2.50, *P* <0.001; AG vs. GG: OR = 1.35, 95% CI = 1.11–1.65, *P* = 0.003) and in hospital-based (HB) controls (A vs. G: OR = 2.15, 95% CI = 1.41–3.29, *P* <0.001; AA/AG vs. GG: OR = 3.15, 95% CI = 1.59–6.21, *P* =0.001; AA vs. GG: OR = 3.20, 95% CI = 1.52–6.76, *P* =0.002; AG vs. GG: OR = 2.95, 95% CI = 1.82–4.79, *P* <0.001) except for the recessive model.

**Table 3:** Pooled ORs and 95% CIs of this meta-analysis for the effect of IL-17A rs2275913 and IL-17F rs763780 polymorphism on colorectal cancer

	Allele model				Dominant model				Recessive model				Homozygous model				Heterozygous model			
	*OR* (95% *CI*)	*P*	*P*_h_	*I*^2^(%)	*OR* (95% *CI*)	*P*	*P*_h_	*I*^2^(%)	*OR* (95% *CI*)	*P*	*P*_h_	*I*^2^ (%)	OR(95% CI)	*P*	P_h_	I^2^(%)	OR(95% CI)	P	P_h_	I^2^(%)
**IL-17A rs2275913 (G197A)**	**A vs. G**				**AA/AG vs. GG**				**AA vs. GG/AG**				**AA vs. GG**				**AG vs. GG**			
**Total**	1.59 (1.34, 1.89)*	< 0.001	0.035	53.5	1.75 (1.36, 2.25)*	< 0.001	0.018	58.5	1.74 (1.41, 2.15)*	< 0.001	0.437	0	2.05 (1.62, 2.60)*	< 0.001	0.117	39.4	1.60 (1.23, 2.09)*	0.001	0.020	58.0
**Ethnicity**																				
Asian	1.52 (1.16, 2.01)*	0.003	0.067	63.0	1.62 (1.18, 2.23)*	0.003	0.186	40.6	1.72 (1.26, 2.34)*	0.001	0.131	50.8	2.10 (1.49, 2.96)*	< 0.001	0.078	60.8	1.43 (1.14, 1.80)*	0.002	0.516	0
Caucasian	1.67 (1.30, 2.14)*	< 0.001	0.058	56.2	1.88 (1.26, 2.81)*	0.002	0.009	70.3	1.76 (1.32, 2.36)*	< 0.001	0.585	0	2.01 (1.46, 2.77)*	< 0.001	0.171	37.5	1.76 (1.11, 2.80)*	0.017	0.004	73.9
**Genotyping method**																				
PCR-RFLP	1.47 (1.24, 1.74)*	< 0.001	0.156	39.8	1.61 (1.23, 2.11)*	0.001	0.075	52.9	1.68 (1.33, 2.14)*	< 0.001	0.219	30.4	1.90 (1.46, 2.47)*	< 0.001	0.172	37.4	1.50 (1.08, 2.08)*	0.015	0.028	63.2
qRT-PCR	2.04 (1.30, 3.20)*	0.002			2.22 (1.27, 3.88)*	0.005			2.26 (0.90, 5.69)	0.084			2.83 (1.10, 7.29)*	0.031			2.03 (1.09, 3.76)*	0.025		
TaqMan	1.84 (0.90, 3.76)	0.092	0.017	82.5	2.42 (0.68, 8.68)	0.174	0.010	85.0	1.88 (1.10, 3.19)*	0.020	0.400	0	2.71 (1.44, 5.08)*	0.002	0.054	73.1	2.17 (0.69, 6.86)	0.186	0.036	77.1
**Source of controls**																				
PB	1.47 (1.25, 1.72)*	< 0.001	0.138	40.1	1.50 (1.23, 1.83)*	< 0.001	0.197	31.8	1.74 (1.39, 2.18)*	< 0.001	0.465	0	1.96 (1.53, 2.50)*	< 0.001	0.260	23.1	1.35 (1.11, 1.65)*	0.003	0.247	25.0
HB	2.15 (1.41, 3.29)*	< 0.001	0.200	39.0	3.15 (1.59, 6.21)*	0.001	0.182	43.9	1.77 (0.98, 3.21)	0.060	0.129	56.5	3.20 (1.52, 6.76)*	0.002	0.053	73.2	2.95 (1.82, 4.79)*	< 0.001	0.408	0
**IL-17F rs763780 (T7488C)**	**C vs. T**				**CC/CT vs. TT**				**CC vs. TT/CT**				**CC vs. TT**				**CT vs. TT**			
**Total**	0.94 (0.63, 1.41)	0.776	0.009	67.5	0.96 (0.64, 1.44)	0.845	0.058	56.2	0.71 (0.45, 1.12)	0.141	0.180	36.2	0.78 (0.32, 1.92)	0.588	0.104	51.3	1.01 (0.79, 1.29)	0.929	0.491	0
**Ethnicity**																				
Asian	1.21 (0.73, 2.01)	0.466	0.067	62.9	1.17 (0.72, 1.89)	0.527	0.142	48.8	1.07 (0.53, 2.18)	0.844	0.076	68.2	1.80 (0.20, 15.89)	0.598	0.056	72.7	1.01 (0.77, 1.32)	0.964	0.300	17.0
Caucasian	0.71 (0.36, 1.36)	0.317	0.043	68.2	0.67 (0.27, 1.63)	0.376	0.055	72.8	0.54 (0.30, 0.98)*	0.042	0.421	0	0.43 (0.21, 0.87)*	0.019	0.694	0	1.03 (0.58, 1.85)	0.910		
**Genotyping method**																				
PCR-RFLP	0.89 (0.54, 1.46)	0.649	0.002	79.3	0.91 (0.58, 1.43)	0.683	0.040	64.0	0.67 (0.41, 1.09)	0.109	0.125	47.8	0.78 (0.32, 1.92)	0.588	0.104	51.3	0.99 (0.76, 1.27)	0.918	0.385	0
qRT-PCR	1.00 (0.33, 3.03)	0.996							1.00 (0.33, 3.09)	0.996										
TaqMan	1.34 (0.56, 3.23)	0.507			1.41 (0.55, 3.59)	0.477											1.41 (0.55, 3.59)	0.477		
**Source of controls**																				
PB	0.94 (0.57, 1.55)	0.804	0.004	73.6	0.95 (0.56, 1.63)	0.854	0.029	66.7	0.71 (0.45, 1.12)	0.146	0.099	52.1	0,83 (0.29, 2.40)	0.733	0.046	67.5	1.01 (0.77, 1.32)	0.964	0.300	17.0
HB	1.01 (0.60, 1.69)	0.976			1.02 (0.58, 1.81)	0.937			0.73 (0.04, 11.78)	0.823			0.73 (0.05,11.94)	0.828			1.03 (0.58, 1.85)	0.910		

*N*, Number of studies included, *OR*, Odds ratio, *CI*, Confidence interval, *P*_h_, *p*-value for heterogeneity. *OR with statistical significance, *P* < 0.05 was considered statistically significant

### Associations between the IL-17F rs763780 polymorphism and colorectal cancer

**Fig. 3:**
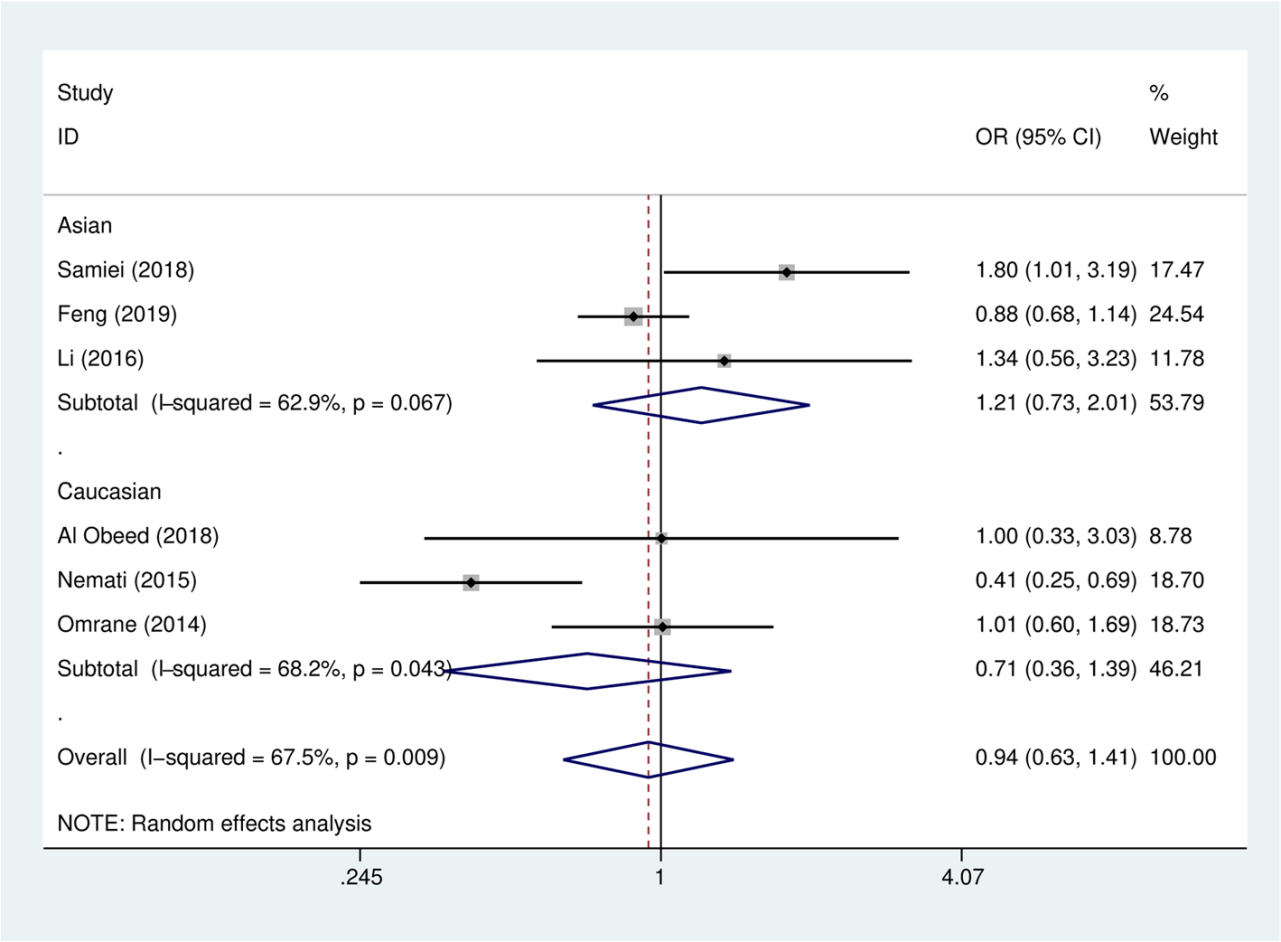
The forest plot of the allelic model (C vs. T) for the associations between IL-17F rs763780 polymorphism and colorectal cancer. The study-specific ORs are represented as squares. The size of the square indicates the weight of each study. The horizontal lines represent 95% CIs. Diamonds show the overall estimate or pooled ORs in subgroups with their corresponding 95% CIs

No significant associations between the IL-17F rs763780 polymorphism and colorectal cancer were detected in the overall analysis (Table 3). We also failed to find any correlations in further subgroup analyses based on the source of controls and genotyping methods. Interestingly, when stratified analysis was classified by ethnicity (Fig. 3, Supplementary Fig. 2 A–D), we discovered a decreased colorectal cancer risk for the Caucasian population in the recessive model (CC vs. CT/TT: *OR* = 0.54, 95% *CI* = 0.30–0.98, *P* =0.042) and homozygous model (CC vs. TT: *OR* = 0.43, 95% *CI* = 0.21–0.87, *P* = 0.019).

### Sensitivity analysis and cumulative meta-analysis

The goal of the sensitivity analysis was to detect whether the pooled OR results could be affected by any single enrolled study. We found no significant alteration in the pooled OR for the IL-17A rs2275913 and IL-17F rs763780 polymorphisms when any one study was eliminated from this meta-analysis, indicating the reliability of our results. The cumulative analysis was performed on the basis of the publication year of the literature, and the results showed that as the number of studies increased, the combined effect sizes and confidence intervals tended to be stable.

### Publication bias

**Fig. 4:**
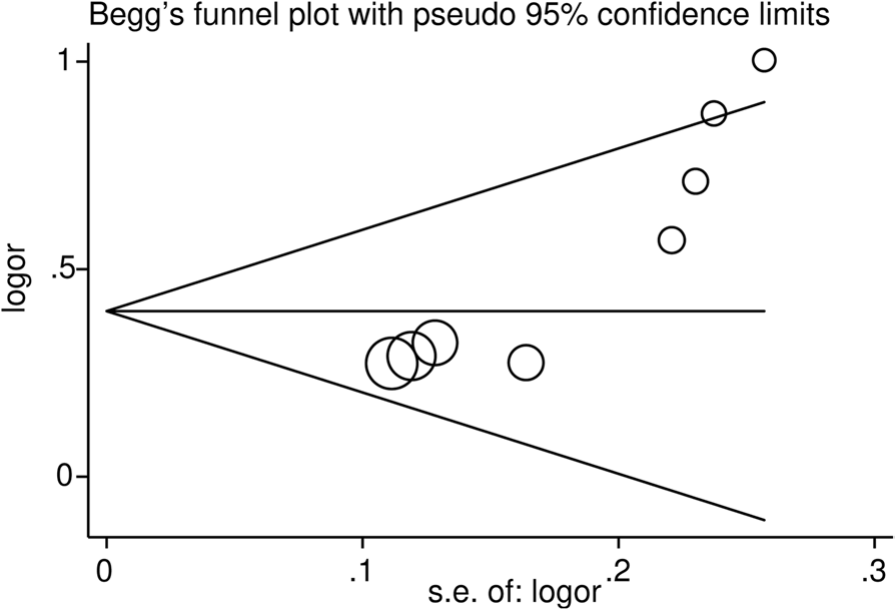
The funnel plot performed to detect the publication bias of included studies regarding IL-17A rs2275913 polymorphism in the allelic model (A vs. G). Each cycle represents an individual case-control study

**Fig. 5:**
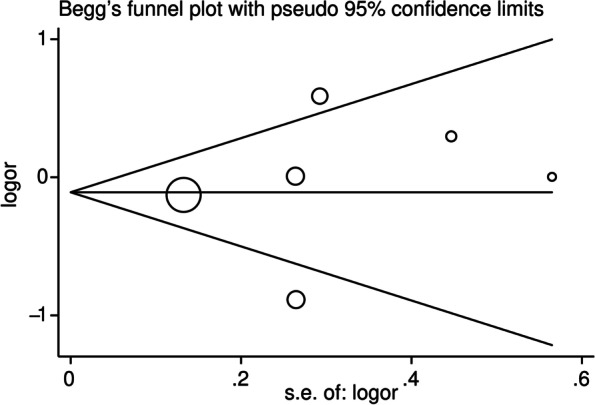
The funnel plot performed to detect the publication bias of included studies regarding to IL-17F rs763780 polymorphism in the allelic model (C vs. T). Each cycle represents an individual case-control study

For the assessment of publication bias, Begg’s funnel plot and Egge’s test were conducted (Fig. 4, Supplementary Fig. 3 A–D). The results for the IL-17A rs2275913 polymorphism displayed a certain publication bias in the allelic model (*P* = 0.001) (Fig. 4) and dominant model (*P* = 0.001) (Supplementary Fig. 3A), and a slight publication bias was observed in the heterozygous model (*P* = 0.021) (Supplementary Fig. 3D). Regarding the IL-17F rs763780 polymorphism, the funnel plots for all of the models were symmetrical and suggested the absence of significant publication bias (Fig. 5, Supplementary Fig. 4 A–D).

## Discussion

Growing evidence has revealed a positive influence of the inflammatory cytokine IL-17 on colorectal cancer development, leading to a poor prognosis for patients. Further studies explicitly determined that IL-17 is involved in colorectal cancer cell proliferation [37], migration and invasion [38], angiogenesis [39], and enhanced drug resistance [40, 41] by regulating a series of downstream signaling pathways, significantly improving the tumorigenesis, invasive and distant metastasis capabilities of colorectal cancer. The role of IL-17 in the occurrence of colorectal cancer has also received increasing attention.

Among the six members of the IL-17 family, IL-17F shared the most similar amino acid sequence and overlapping functions with IL-17A [42]. Each of the two genes consisted of 3 exons and 2 introns and was located on chromosome 6p12.3-q13. The genetic variant of IL-17A rs2275913 was located in the 5′-UTR, which is involved in gene transcription regulation and changes the roles of some cytokines [43]. The IL-17F rs763780 polymorphism was identified as a missense mutation located in the coding region, with the amino acid modification of the conversion of His to Arg, resulting in potential changes in protein expression and possible cancer risk [30]. An increasing number of studies and meta-analyses have been performed to explore associations between the IL-17A rs2275913 and IL-17F rs763780 polymorphisms and various types of malignant tumors in recent years [44–46]; however, the related findings for colorectal cancer display no consensus. Thus, this meta-analysis was performed to detect whether both polymorphisms contribute to colorectal cancer susceptibility.

Our present research was comprised of 2599 cases and 2845 controls from the 10 selected case-control studies. The overall analysis results revealed highly significantly positive associations between the IL-17A rs2275913 polymorphism and colorectal cancer in all five genetic models (A vs. G, AA/AG vs. GG, AA vs. AG/GG, AA vs. GG, and AG vs. GG), suggesting that this mutation may be a remarkable genetic risk factor in the tumorigenesis of colorectal cancer. However, when the analysis was performed for the IL-17F rs763780 polymorphism, no associations for colorectal cancer were observed in any genetic models (C vs. T, CC/CT vs. TT, CC vs. CT/TT, CC vs. TT, and CT vs. TT). The combined effect size did not change significantly when the enrolled studies were excluded one by one, ensuring the reliability of these associations. In addition, it was noteworthy that heterogeneities existed in the statistical results for some genetic models.

To explore the origin of heterogeneities and further explain the impact of different factors on the contributions of IL-17A rs2275913 and IL-17F rs763780 polymorphisms to colorectal cancer susceptibility, a series of subgroup analyses based on the aspects of race, source of controls, and genotyping method were conducted. The results of the analysis classified by ethnicity displayed an increased colorectal cancer risk from the IL-17A rs2275913 polymorphism in both the Asian and Caucasian subgroups, revealing that this mutation might independently increase the susceptibility to colorectal cancer risk in Asian and Caucasian populations. However, for the IL-17F rs763780 polymorphism, a decreased risk correlated with colorectal cancer in the Caucasian subgroup was observed in the recessive and homozygous models, which suggested that the biological functions of the IL-17F rs763780 polymorphism for populations from various races were possibly discrepant and provided a negative predictor for colorectal cancer occurrence in Caucasians; however, due to the insufficient sample size, such a result needs to be identified by further studies. When stratified analysis was performed in terms of the source of controls, we found that only the HB population in the recessive model of the IL-17A rs2275913 polymorphism showed no significant relationship with elevated colorectal cancer risk. Since patients with self-underlying diseases were included, potential selection bias was likely to decrease the representativeness of controls in the HB group compared to those in the PB group [47]. In the models of the IL-17F rs763780 polymorphism, no significant association with colorectal cancer susceptibility was observed in either PB or HB populations. We further discovered some statistical discrepancies among the subgroups divided by genotyping methods for the IL-17A rs2275913 polymorphism. The explanation may be that various gene detection methods have different theories and advantages, which possibly lead to different testing results [48].

Although this meta-analysis was performed with rigorous design and exact calculations, several inevitable limitations should be noted. First, some heterogeneities were observed in the overall analyses for both polymorphisms, and stratified analyses classified by ethnicity, the source of controls, and some other subgroups failed to completely eliminate these heterogeneities. Second, systematic reviews using case-control studies are prone to error of inappropriate selection of control groups for comparison. The data of age, sex, living styles, and exposures to smoking or drinking were unable to be further extracted, and since such factors may also impact the occurrence and development of cancer, available information of these unadjusted estimates was essential for a more accurate analysis. Third, all of the selected studies were conducted in Asian and Caucasian populations, and the geographic areas were limited to East Asia, West Asia, and North Africa. Such geographical bias might be attributed to more attention given to colorectal cancer prevention due to the substantially increased colorectal cancer incidence in Arab and eastern Asian countries during recent years [49–52]. In addition, a candidate gene association study was a cost-effective and convenient hypothesis-driven approach, which may make it readily available to investigate genetic susceptibility to colorectal cancer in these countries [53]. Therefore, studies with related data from other races and geographic areas are required to verify these findings. Fourth, all of the included literature was published in English and Chinese, and papers written in other languages and unpublished data due to negative results were not obtained, which may be responsible for the publication bias detected in the IL-17A rs2275913 polymorphism. Future analysis with more enrolled studies would likely overcome this issue. Moreover, the sample size of this meta-analysis was relatively small, and the findings need to be discussed in further studies with large samples.

Admittedly, the results should be interpreted with caution due to these limitations, but some possible benefits of our present study may be worthy of attention. First, to the best of our knowledge, this was the first meta-analysis to specifically detect the relationships between IL-17 polymorphisms and colorectal cancer risk. Furthermore, in this study, we sought to systematically evaluate previous studies by a meta-analysis to obtain reliable conclusions about the association of IL-17A rs2275913 and IL-17F rs763780 polymorphisms with susceptibility to colorectal cancer, which probably provides a new perspective for mechanistic research and a novel direction for the clinical treatment of this life-threatening malignancy. Finally, the potential uses of our findings also included earlier screening and family genetic testing for identifying high-risk patients.

In conclusion, this meta-analysis displayed a significant association between the IL-17A rs2275913 polymorphism and susceptibility to colorectal cancer among Asians and Caucasians, which provided a potential risk factor for colorectal cancer for the two populations. Although we failed to discover any positive effects of the IL-17F rs763780 polymorphism on colorectal cancer occurrence, this mutation may decrease the colorectal cancer risk in Caucasians. The analysis results shown in our present research should be confirmed by continued well-designed and high-level studies, especially some prospective studies, in the future.

## Supplementary Information

**Additional file 1: Supplementary Figure 1.** Forest plots of the genetic models for the associations between IL-17A rs2275913 polymorphism and colorectal cancer. **A**. dominant model (AA/AG vs. GG). **B**. recessive model (AA vs. GG/AG). **C**. homozygous model (AA vs. GG). **D**. heterozygous model (AG vs. GG). The study-specific ORs are represented as squares. The size of the square indicates the weight of each study. The horizontal lines represent 95% CIs. Diamonds show the overall estimate or pooled ORs in subgroups with their corresponding 95% CIs.

**Additional file 2: Supplementary Figure 2.** Forest plots of the genetic models for the associations between IL-17F rs763780 polymorphism and colorectal cancer. **A.** dominant model (CC/CT vs. TT)**. B.** recessive model (CC vs. TT/CT). **C.** homozygous model (CC vs. TT)**. D.** heterozygous model (CT vs. TT). The study-specific ORs are represented as squares. The size of the square indicates the weight of each study. The horizontal lines represent 95% CIs. Diamonds show the overall estimate or pooled ORs in subgroups with their corresponding 95% CIs.

**Additional file 3: Supplementary Figure 3.** Funnel plots performed to detect the publication bias of included studies regarding to IL-17A rs2275913 polymorphism in the genetic models. **A**. dominant model (AA/AG vs. GG). **B**. recessive model (AA vs. GG/AG). **C**. homozygous model (AA vs. GG). **D**. heterozygous model (AG vs. GG). Each cycle represents an individual case-control study.

**Additional file 4: Supplementary Figure 4.** Funnel plots performed to detect the publication bias of included studies regarding to IL-17F rs763780 polymorphism in the genetic models. **A.** dominant model (CC/CT vs. TT)**. B.** recessive model (CC vs. TT/CT). **C.** homozygous model (CC vs. TT)**. D.** heterozygous model (CT vs. TT). Each cycle represents an individual case-control study.

## Data Availability

The datasets used and/or analyzed during the current study are available from the corresponding author on reasonable request.

## Abbreviations

IL-17: Interleukin-17
CBM: Chinese Biomedical Literature Database
CNKI: China National Knowledge Infrastructure
OR: Odds ratio
CI: Confidence interval
HWE: Hardy–Weinberg genetic equilibrium law
PB: Population based
HB: Hospital based
