# Supplementary figures and images for: The associations between interleukin-17 single-nucleotide polymorphism and colorectal cancer susceptibility: a systematic review and meta-analysis
Source: World J Surg Oncol. 2022 Apr 12;20:116. doi: 10.1186/s12957-022-02586-2 (PMC9004118; doi:10.1186/s12957-022-02586-2)

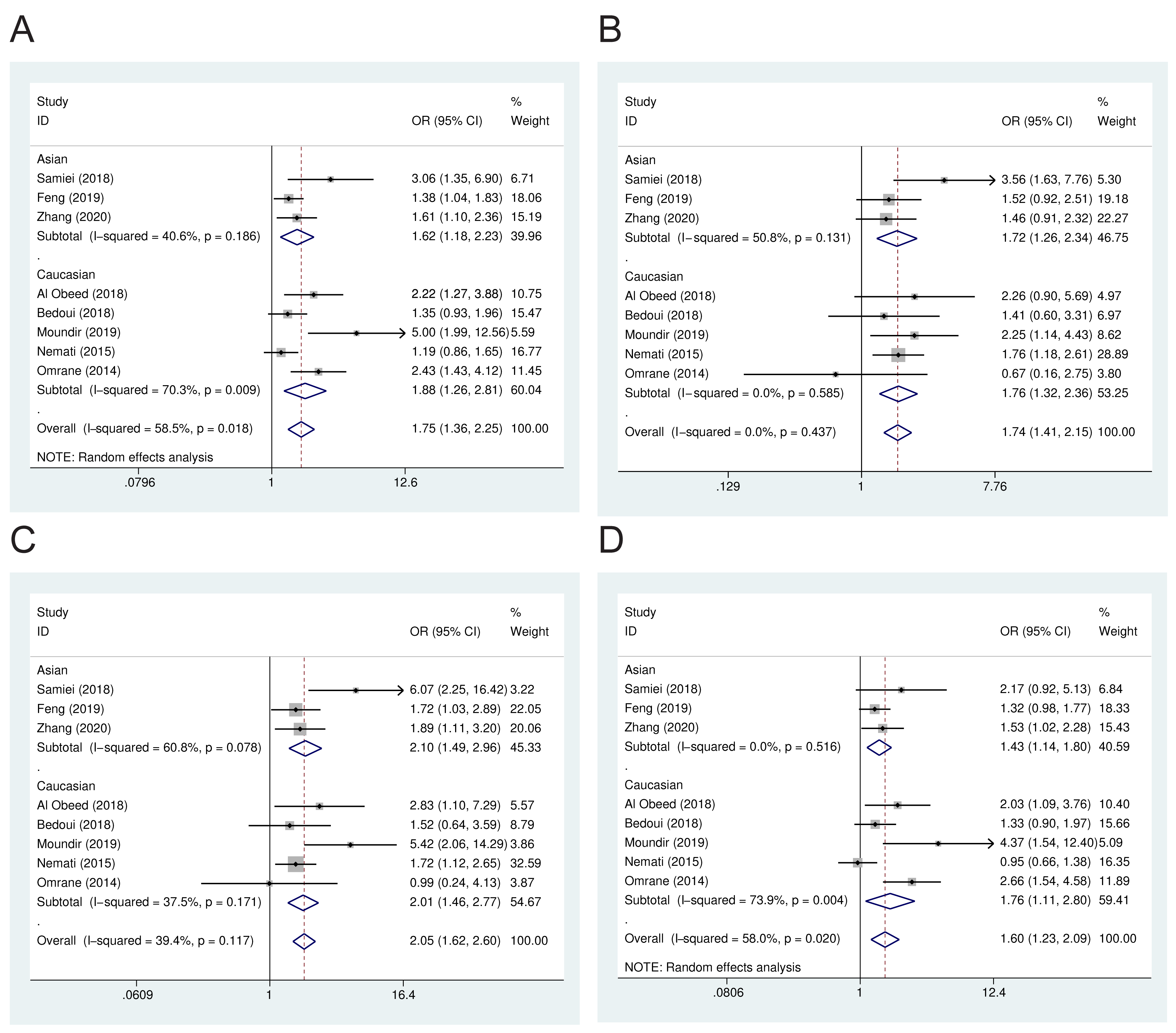

Supplement: Supplementary file 1 — Additional file 1: Supplementary Figure 1. Forest plots of the genetic models for the associations between IL-17A rs2275913 polymorphism and colorectal cancer. A. dominant model (AA/AG vs. GG). B. recessive model (AA vs. GG/AG). C. homozygous model (AA vs. GG). D. heterozygous model (AG vs. GG). The study-specific ORs are represented as squares. The size of the square indicates the weight of each study. The horizontal lines represent 95% CIs. Diamonds show the overall estimate or pooled ORs in subgroups with their corresponding 95% CIs. [file 12957_2022_2586_MOESM1_ESM.tif]

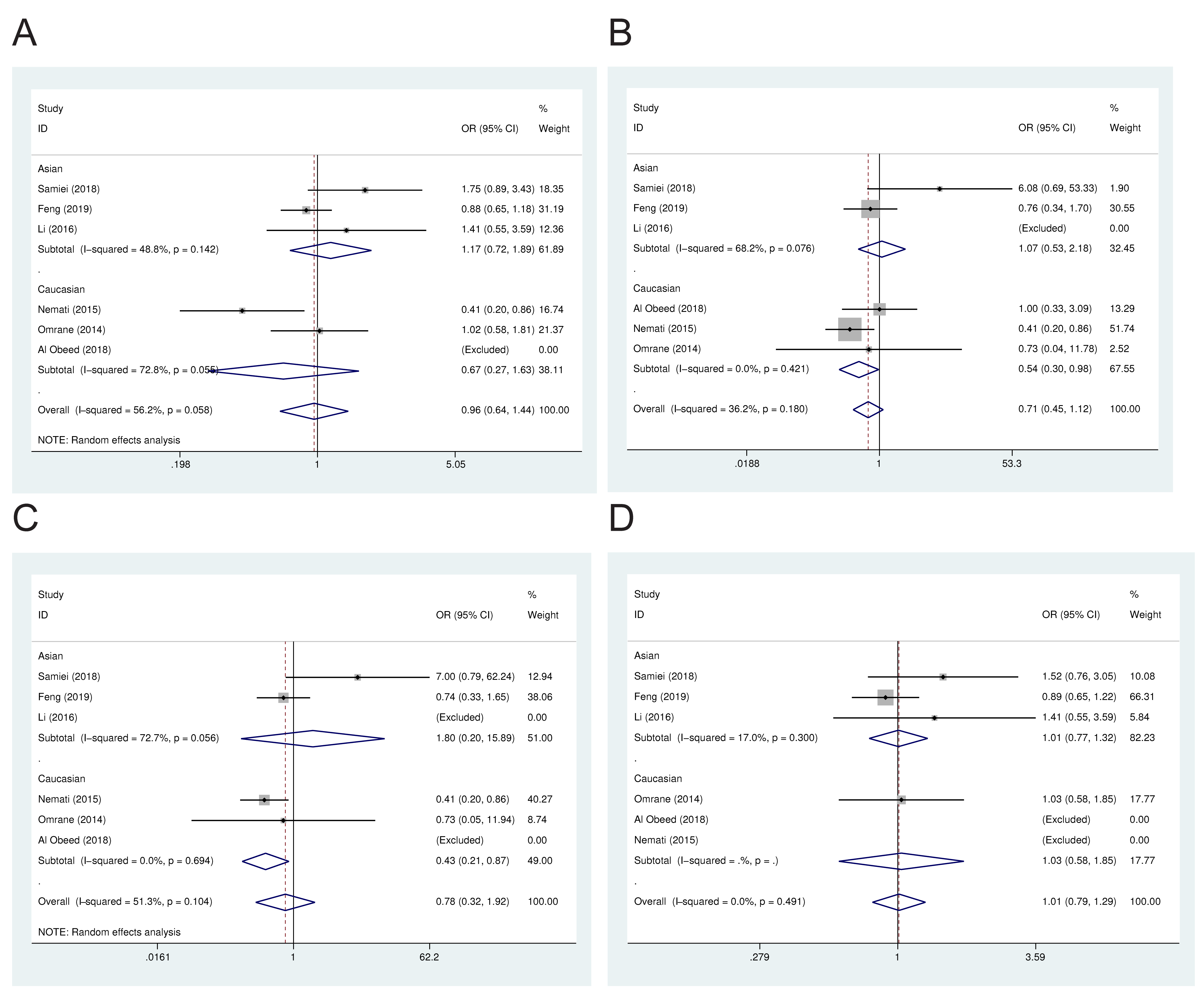

Supplement: Supplementary file 2 — Additional file 2: Supplementary Figure 2. Forest plots of the genetic models for the associations between IL-17F rs763780 polymorphism and colorectal cancer. A. dominant model (CC/CT vs. TT). B. recessive model (CC vs. TT/CT). C. homozygous model (CC vs. TT). D. heterozygous model (CT vs. TT). The study-specific ORs are represented as squares. The size of the square indicates the weight of each study. The horizontal lines represent 95% CIs. Diamonds show the overall estimate or pooled ORs in subgroups with their corresponding 95% CIs. [file 12957_2022_2586_MOESM2_ESM.tif]

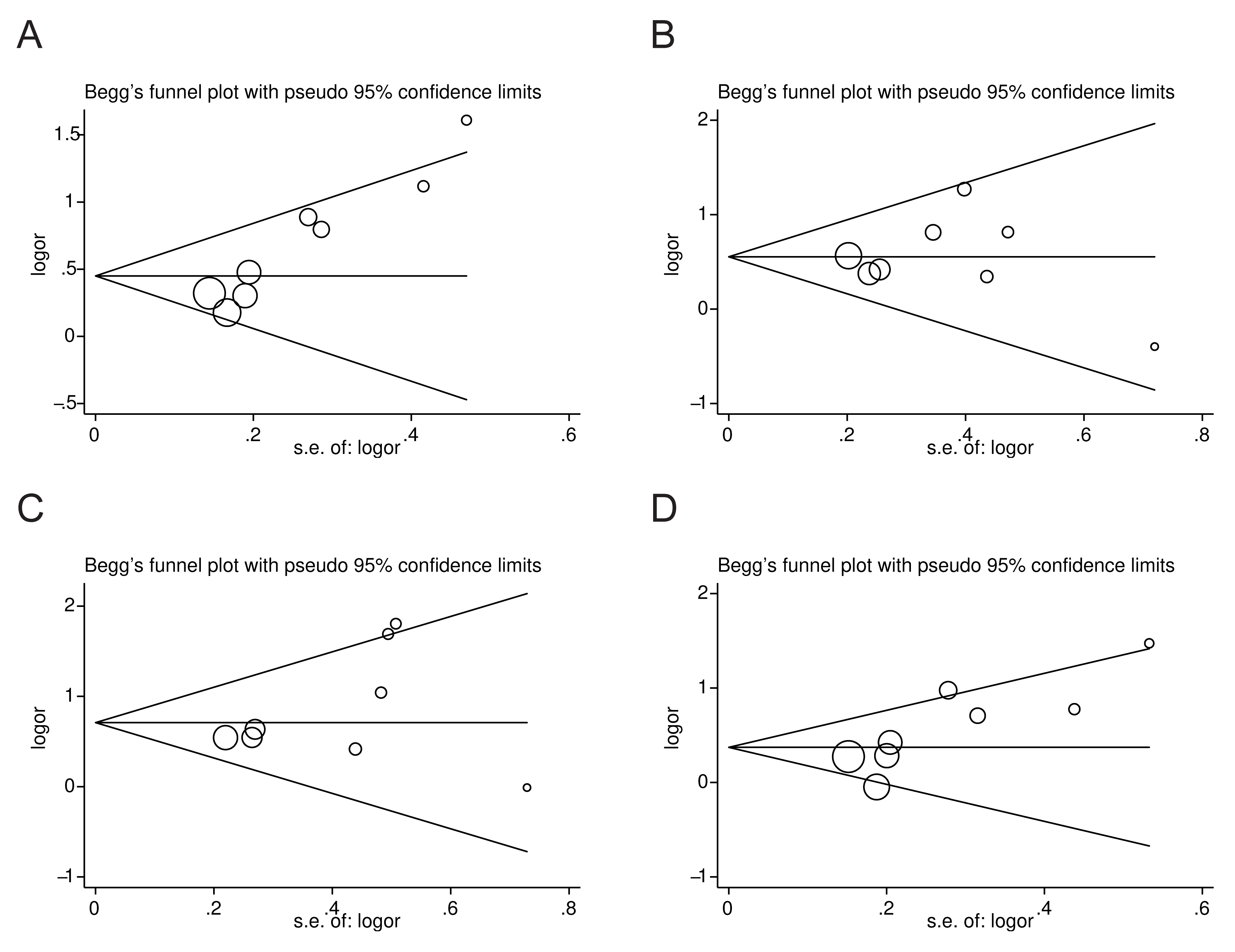

Supplement: Supplementary file 3 — Additional file 3: Supplementary Figure 3. Funnel plots performed to detect the publication bias of included studies regarding to IL-17A rs2275913 polymorphism in the genetic models. A. dominant model (AA/AG vs. GG). B. recessive model (AA vs. GG/AG). C. homozygous model (AA vs. GG). D. heterozygous model (AG vs. GG). Each cycle represents an individual case-control study. [file 12957_2022_2586_MOESM3_ESM.tif]

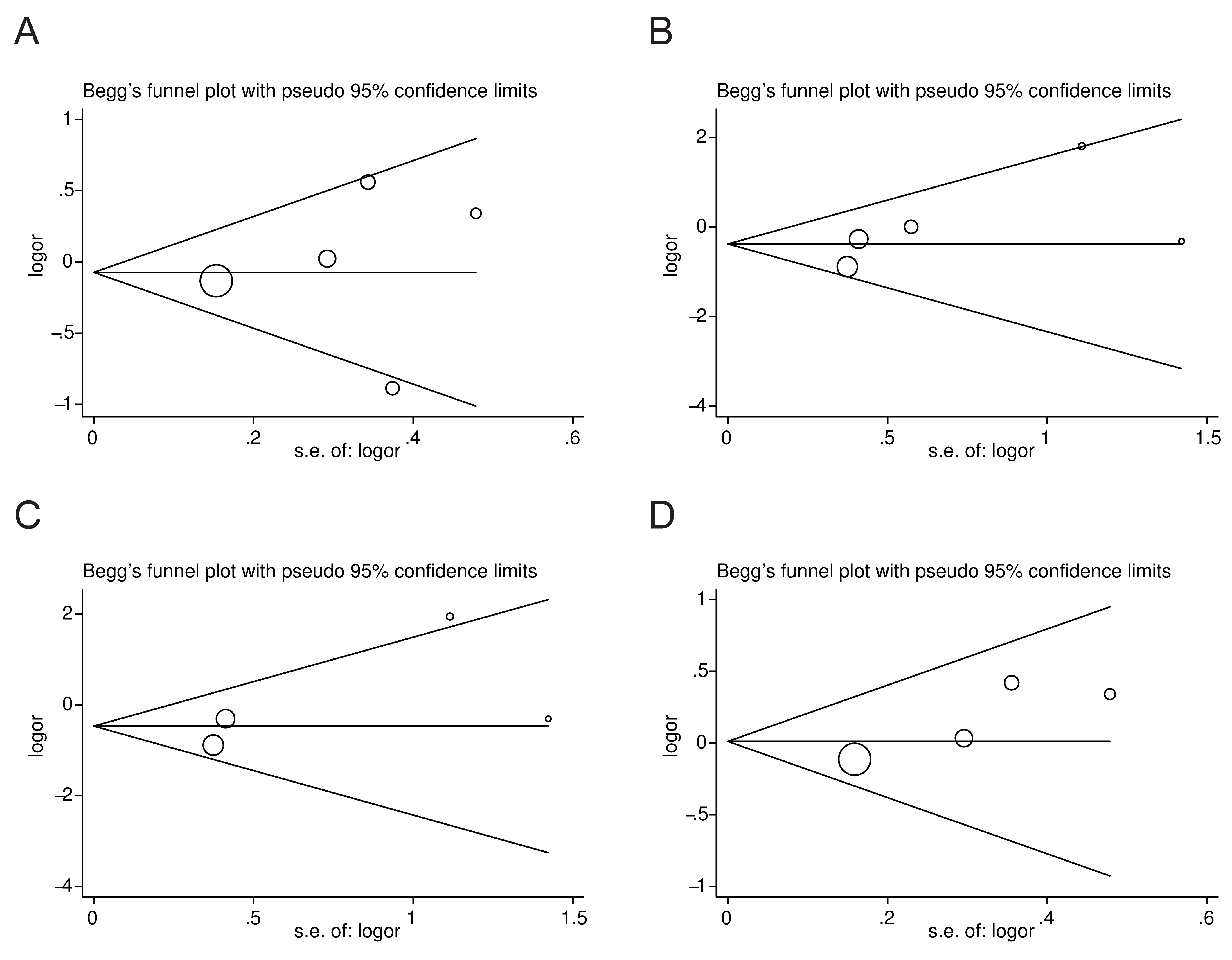

Supplement: Supplementary file 4 — Additional file 4: Supplementary Figure 4. Funnel plots performed to detect the publication bias of included studies regarding to IL-17F rs763780 polymorphism in the genetic models. A. dominant model (CC/CT vs. TT). B. recessive model (CC vs. TT/CT). C. homozygous model (CC vs. TT). D. heterozygous model (CT vs. TT). Each cycle represents an individual case-control study. [file 12957_2022_2586_MOESM4_ESM.tif]
